# The complete chloroplast genome of *Agropyron desertorum* (Fisch. ex Link) Schult

**DOI:** 10.1080/23802359.2021.1938721

**Published:** 2021-06-14

**Authors:** Junfeng Yang, Wenxuan Du, Yongzhen Pang

**Affiliations:** a Institute of Animal Sciences, Chinese Academy of Agricultural Sciences, Beijing, China; b Key Laboratory of Plant Resources and Beijing Botanical Garden, Institute of Botany, Chinese Academy of Sciences, Beijing, China; c University of Chinese Academy of Sciences, Beijing, China

**Keywords:** *Agropyron desertorum* (Fisch. ex Link) Schult., chloroplast genome, phylogenetic analysis

## Abstract

*Agropyron desertorum* is one of the most important fodder grasses which distributes in the temperate regions of the world. In this study, the complete chloroplast genome of *A. desertorum* was sequenced. The genome was 135,459 bp in length, with a large single-copy region of 79,617 bp, a small single-copy region of 12,760 bp, and two inverted repeat regions of 21,541 bp. The genome consisted of 132 genes, including 86 protein-coding genes, 38 tRNA genes, and 8 rRNA genes. The GC contents was 38.32%. We constructed the Maximum likelihood (ML) tree with 13 species from the Hordeinae genus, and concluded that *A. desertorum* was closely related to plant species of the *Elymus* genus in the phylogenetic tree.

*Agropyron desertorum* (Fisch. ex Link) Schult. belongs to the Gramineae family, which is one of the most important grasses in the temperate regions of the world. *A. desertorum* is a wild grass with strong growth potential, high yield, soft quality and high nutrition value, which is an excellent source of forage and habitat for livestock and wildlife (Jafari et al. 2008). *A. desertorum* has strong drought resistance, cold resistance and sand fixation function, which is valued for weed control, habitat use, soil conservation and watershed management. *Agropyron desertorum* is generally adapted to sub-humid to arid climatic conditions in steppe or desert regions (Jafari et al. 2008). Due to these valuable characteristics, *A. desertorum* is suitable for the restoration and rehabilitation of rangelands in the arid and semi-arid regions (Goraghani et al. 2013; Shanjani et al. 2013). In this study, we determined the complete chloroplast genome sequences of *A. desertorum* and compared the sequence with those of other species of Hordeinae to analyze the phylogenetic relationship among them.

Seeds of *A. desertorum* was kept at the Forage Germplasm Bank at Institute of Animal Sciences of the Chinese Academy of Agricultural Sciences (Beijing, E116°29′, N40°03′). The voucher specimen (FG17198) was deposited at the Herbarium of the Institute of Animal Sciences of the Chinese Academy of Agricultural Sciences, Beijing, China. After germination in the laboratory, genomic DNA from young leaves was extracted using a DNA Extraction Kit from Tiangen Bio Tech Co., Ltd (Beijing, China). The genomic DNA was broken and fragmented to 400 bp fragments by using the high-pressure nitrogen or a Covaris machine, and these fragments were used as the template for the paired-end library. The paired-end library was constructed with Illumina TruSeq DNA Sample Preparation kit according to the Illumina TruSeq DNA Sample Preparation Guide. The sequencing was carried out on the Illumina Novaseq PE150 platform (Illumina Inc, San Diego), and 150 bp paired-end reads were generated. The software GetOrganelle v1.5 (Jin et al. 2018) was used to assemble the cleaned reads into a complete chloroplast genome with the chloroplast genome of *Alopecurus aequalis* (NC_047228.1) as reference. The chloroplast genome annotation was performed through the online program CPGAVAS2 (Shi et al. 2019) and GeSeq (Tillich et al. 2017), followed by manual correction. The assembled chloroplast genome sequence was submitted to GenBank under the accession number MW309818.

In the present study, it was found that the chloroplast genome of *A. desertorum* showed a quadripartite structure, with two reverse repeated regions (IRa and IRb) of 21,541 bp in length. The repeat regions divided the genome into two single-copy regions, SSC and LSC with12,760 bp and 79,617 bp, respectively. The GC contents of the LSC, SSC, and IR regions individually, and of the chloroplast genome as a whole, are 39.64%, 35.72%, 36.66%, and 38.32%, respectively. It encodes a total of 132 unique genes, of which 42 are duplicated in the IR regions. Out of the 132 genes, there are 86 protein-coding genes, 38 tRNA genes, and 8 rRNA genes. Nineteen genes contained introns, and 17 (nine protein-coding and eight tRNA genes) of them contains one intron and two of them (*rps12* and *ycf3*) contain two introns.

The chloroplast genomes of 13 species from Hordeinae were downloaded from the NCBI GenBank database to inference the phylogenetic relationship of *A. desertorum*. The sequences were aligned using MAFFT v7 (Katoh et al. 2019). In addition, a maximum likelihood (ML) tree based on the common protein-coding genes of 14 species, with *Avena sativa* as out-group, was constructed by using raxmlGUI1.5b (v8.2.12) (Silvestro and Michalak 2012). Phylogenetic analysis shows that *A. desertorum* is closely related to the plant species of the *Elymus* genus (e.g. *E. trachycaulus*, *E. dahuricus* and *E. kamoji*) in the phylogenetic tree (Figure 1). This study will provide important information for species identification and phylogenetic relationship in the Gramineae family, in particular for Hordeinae forage.

**Figure 1.**
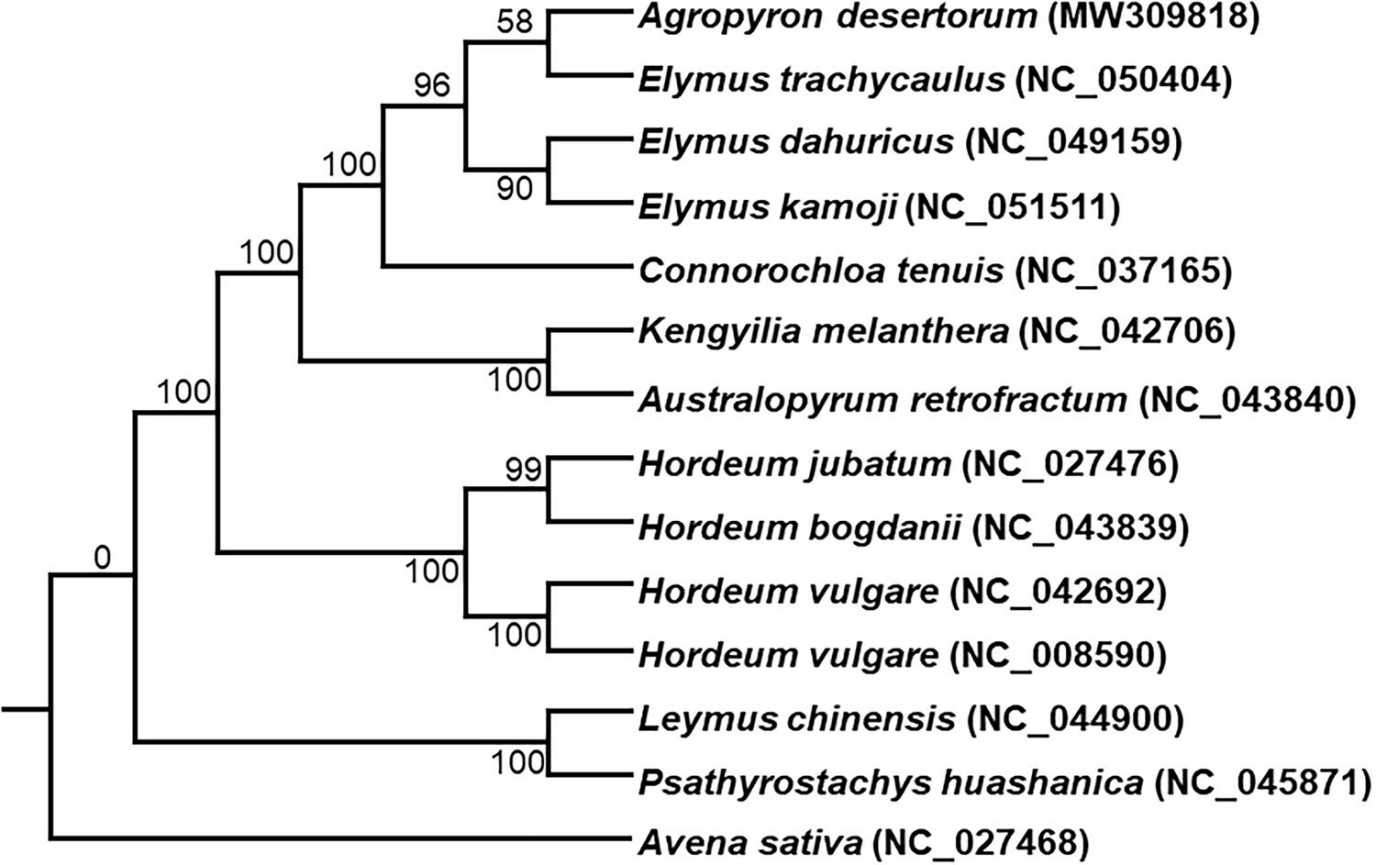
Phylogenetic tree constructed using maximum likelihood (ML) method based on the common protein-coding genes of 13 species of the Gramineae family, with *Avena sativa* as the out-group by using raxmlGUI1.5b and the best nucleotide substitution model: PROTGAMMAILGX. Numbers above the nodes are bootstrap values based on 1000 replicates.

## Data Availability

The data that support the findings of this study are openly available in NCBI at GenBank with accession number MW309818 (https://www.ncbi.nlm.nih.gov/nuccore/MW309818). Raw sequencing reads used in this study was deposited in the public repository SRA with accession number SRR13495514 (https://www.ncbi.nlm.nih.gov/sra/?term=SRR13495514).
